# Viral diversity, ecological interconnectedness, and the identification of mammalian chuviruses in Australian microbats

**DOI:** 10.1093/ve/veag017

**Published:** 2026-03-21

**Authors:** Ayda Susana Ortiz-Baez, Julien Mélade, Kate Van Brussel, Bethan J Lang, Anna Lachenauer, Edward C Holmes

**Affiliations:** School of Medical Sciences, University of Sydney, Sydney, New South Wales 2006, Australia; School of Medical Sciences, University of Sydney, Sydney, New South Wales 2006, Australia; School of Medical Sciences, University of Sydney, Sydney, New South Wales 2006, Australia; School of Medical Sciences, University of Sydney, Sydney, New South Wales 2006, Australia; Department of Dermatology, Stanford University School of Medicine, Redwood City, CA 94063, United States; School of Medical Sciences, University of Sydney, Sydney, New South Wales 2006, Australia

**Keywords:** bat, virus, evolution, ecology, Australia, coronavirus, chuvirus

## Abstract

Microbats are a large and ecologically important group of Australian mammalian fauna. However, their RNA virome diversity, as well as its ecological and evolutionary significance, has received limited study. We applied a metatranscriptomic approach to reveal more of the diversity of RNA viruses present in faeces from different microbat species in New South Wales and South Australia, including the critically endangered Southern bent-wing bat (*Miniopterus schreibersii bassanii*) from the Naracoorte bat maternity caves in South Australia. The data generated revealed a high diversity of RNA viruses, including 51 likely mammalian-associated viruses classified into ten taxonomic groups, including the *Coronaviridae*, *Hepeviridae*, and *Chuviridae*. Notably, we identified a mammalian-specific lineage of chuviruses associated with bats in Australia and with bats and rodents in China, strongly suggesting that viruses of this family have established sustained transmission cycles in mammals as well as invertebrates. Our results also revealed widespread viral connectivity among alphacoronaviruses across multiple microbat species in mainland Australia and Christmas Island, indicative of long distance viral movement. High viral diversity and virus co-circulation was observed within the Southern bent-wing bat population of the Naracoorte caves, suggesting complex population dynamics that might facilitate virus maintenance and transmission. Overall, these findings highlight the role of Australian microbats as viral reservoirs, including the presence of viruses not previously associated with sustained mammalian transmission.

## Introduction

Comprising over 1 400 species, Chiroptera (i.e. bats) is one of the most species-rich orders of mammals, ranking second only to Rodentia (de Oliveira 2022). This diversity encompasses a wide range of biological features, including varied diets, body sizes, sensory adaptations, hibernation patterns and roosting behaviours. Microbats, formerly classified as Microchiroptera, are now incorporated into the suborders Yinpterochiroptera and Yangochiroptera, and comprise small-sized, laryngeal echolocating bats (Tsagkogeorga et al. 2013). These animals mainly rely on echolocation for navigating and foraging for insects, although diverse feeding behaviours are found in some species (de Oliveira 2022). Prey composition primarily comprises lepidopterans, dipterans, as well as hemipterans and coleopterans among other arthropod taxa (Arrizabalaga-Escudero et al. 2015, Ancillotto et al. 2023).

Microbats roost in a wide range of habitats, including caves, crevices, and tree cavities (Law et al. 2011). To mitigate habitat loss and human-wildlife interactions, artificial roosting sites such as bat boxes are built to provide shelter to bats in urban and natural settings (Law et al. 2011, Rueegger 2016, Godinho et al. 2020). The occupancy of bat boxes by different species may vary depending on geographic location, seasonal dynamics, and the structural design of the boxes (Rueegger 2016). Depending on environmental conditions and food availability, microbats are also able to reduce their physiological activity and energy demand by entering torpor, especially as a mechanism to cope with low temperatures (Law et al. 2011, Doty et al. 2019). Natural habitats such as caves provide shelter with more stable levels of temperature and humidity, which are essential for entering topor and breeding (Perry 2013, Zukal et al. 2017). For instance, maternity caves are well-suited for resting and nursing newborn bats, thereby supporting colony social structure, and hence contribute to bat conservation and population well-being (Hamilton-Smith 1970, Bourne 2010, Perry 2013, Zukal et al. 2017).

Australia is home to 77 species of bats. While these species vary in their spatial distribution, some exhibit a wide range across the country. For example, the lesser long-eared bat (*Nyctophilus geoffroyi*) and Gould’s wattled bat (*Chalinolobus gouldii*) are endemic to Australia and occur in a wide range of habitats and environments (IUCN 2025). In contrast, species like the large bent-wing bat (*Miniopterus schreibersii oceanensis*) and the little forest bat (*Vespadelus vulturnus*) are restricted to south-eastern Australia. These microbats also exhibit diverse roosting strategies, ranging from solitary behaviour to the formation of large seasonal colonies, with up to 35 000 individuals in the case of the Southern bent-wing bat (*Miniopterus orianae bassanii*) (Lear 2012). Bat congregation is often associated with reproduction, hibernation, mating, or the establishment of maternity colonies.

According to conservation assessments and the IUCN red list (IUCN 2025), many microbat species are experiencing a decrease in population size due to habitat loss, climate change, limited resource availability, and the presence of invasive species (Voigt and Kingston 2015, O’Shea et al. 2016, Frick et al. 2024). Population declines have been documented in Australia, including in the little forest bat, the white-striped free-tailed bat (*Tadarida australis*) and the yellow-bellied shear bat (*Saccolaimus flaviventris*), with the cave-roosting Southern bent-wing bat considered a threatened species (Parish et al. 2012, Woinarski 2018). These declines are largely due to habitat destruction caused by urban development, logging, mining, wildfires and the introduction of invasive species (Hopkins 2015). Aside from population declines, these stressors can trigger downstream effects, including alterations in foraging patterns, metabolic disruption, impaired immune response, and even increased virus shedding (Plowright et al. 2015, Lagergren et al. 2025, Niu and McKee 2025).

Revealing the diversity of viruses in wild microbats provides important information on virus population structure and virus-host associations, and assists in the identification of viruses that may have implications for bat conservation as well as those with zoonotic potential (Field 2018, Prada et al. 2019). Overall, the most common RNA virus families reported in microbats are the *Coronaviridae*, *Rhabdoviridae*, *Astroviridae*, *Picornaviridae* and *Paramyxoviridae* (Prada et al. 2019, Zhou et al. 2022). As bat conservation is paramount, documenting the bat virome represents an important way to identify potential pathogens that could impact the well-being of bat populations or those with zoonotic potential. Fortunately, faecal sampling offers a viable, non-invasive approach for the surveillance of viruses shed in bat guano, including both mammalian-origin viruses and diet-derived viruses (i.e. viruses originated from ingested invertebrate prey) (Van Brussel et al. 2022, Waller et al. 2024).

Although Australia harbours a remarkable diversity of microbats, the composition, evolution, and ecology of their mammalian-associated RNA virome is largely unexplored. Herein, we use a meta-transcriptomics (i.e. total RNA sequencing) approach to analyse faecal samples collected from microbats in natural and artificial roosting sites in New South Wales and South Australia. From the data generated we assess the RNA virus diversity, ecological patterns and virome connectivity among microbat populations.

## Methods

### Ethical approval and scientific licences

The collection of bat faecal samples was conducted under approval from the University of Sydney Animal Ethics Committee (Project number: 2023/AE002279). Fieldwork in South Australia was performed under the relevant permit issued by the Government of South Australia, Department for Environment and Water (Permit number: E27285–3).

### Sample collection

**Figure 1:**
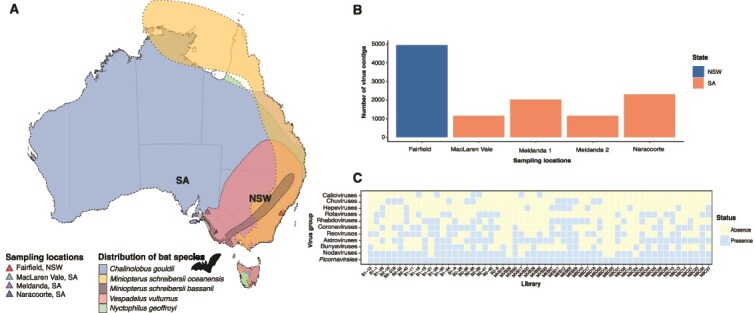
Overview of bat host range, viral contig counts per location and virus prevalence in the bat samples taken. (A) Range distribution of the most common bat species in NSW and SA, Australia. Each species distribution is displayed in a different colour. Sampling locations are indicated with colour coded triangles. Species distributions were accessed from the IUCN and ATLAS web portals [IUCN 2025, Belbin et al. 2021]. (B) Number of virus contigs by sampling location. (C) Distribution of virus groups across the sample set. Each square denotes a single sample. Blue indicates virus presence, yellow denotes absence.

Faecal samples from microbat species were opportunistically collected by placing tarps (1 m x 1.5 m) under roosting sites, including bat boxes, in various locations in New South Wales (Fairfield), and South Australia (Adelaide), Australia. Additional faecal samples were collected from the entrance of the Naracoorte bat maternity caves in south-eastern South Australia (Table 1, Fig. 1). Bat boxes typically had height ~ 40–60 cm and width ~ 15–25 cm and included narrow internal chambers. Colony size estimates were only available for the bat roost in Naracoorte bat maternity cave (at ~ 20 000 to 35 000 individuals). Three to five tarps were placed beneath bat boxes or at designated locations on the cave floor from 5:00 pm–7:00 am to collect faecal pellets dropped during the nightly emergence and upon the bats return to the roosts. To avoid cross-contamination all tarps were single-use. To minimize disturbance to the animals and ensure their welfare, all samples were collected during spring, after the hibernation period. Tarps were quietly deployed and retrieved while bats were at rest to avoid disturbance. The number of samples collected varied, likely reflecting differences in both roost type and number of bats (Table 1). All samples were taken from a single sampling time point, preserved in DNA/RNA Shield and stored at −80 °C to aid RNA integrity and deactivate viruses. Each tube contained three to five faecal pellets.

**Table 1:** Bat sampling locations in new South Wales (NSW) and South Australia (SA), with the expected bat species at each site. Host species confirmed by the presence of mitochondrial transcripts in RNA sequencing data are indicated in boldface. The total number of samples is reported as the number of successful samples out of the total processed.

Number of samples	Location [Abbreviation]	Bat roost	Bat species	Criteria for taxonomic assignment of bat species^&^	Conservation status (IUCN)
22/88	Fairfield, Sydney, NSW [B]	Bat boxes (n = 2)	**Little forest bat (*Vespadelus vulturnus*)** ^**≡**^	(i) and (iii)	Least concern ↓
5/23	Bat island^Δ^, Meldanda reserve, SA [MEB]	Bat boxes (n = 5)	Lesser long-eared bat (*Nyctophilus geoffroyi*)^Ψ^; White-striped free-tailed bat (*Tadarida australis*); Little forest bat (*V. vulturnus*); Chocolate wattled bat (*Chalinolobus morio*); Inland broad-nosed bat (*Scotorepens balstoni*); Southern freetail bat (*Mormopterus sp.*); Yellow-bellied shear bat (*Saccolaimus flaviventris*); Gould’s wattled bat (*Chalinolobus gouldii*)^Ψ^; Southern forest bat (*Vespadelus regulus*); The large forest bat (*Vespadelus darlingtoni*)	(ii) and (iii)	Least concern Least concern ↓ Least concern ↓ Least concern Least concern ↓ Least concern ↓ Least concern Least concern Least concern Least concern
6/22	Property Meldanda, SA [MDB]	Bat boxes (n = 3)	Unknown; possibly similar species present at MEB.	(ii) and (iii)	
16/30	Naracoorte bat caves, SA [NBC]	Maternity cave	Southern bent-wing bat (***Miniopterus schreibersii bassanii****)^¤^*	(i), (ii) and (iii)	Vulnerable (VU)^ω^↓
8/17	McLaren Valley, SA [MVB]	Building Crevices	**Lesser long-eared bat (*Nyctophilus geoffroy*)** ^*^; unknown	(i) and (iii)	Least concern

Ψ Bat species were identified through the assessment of morphological characteristics during the 2013–2018 survey.

Δ Bat Island includes 30 bat boxes supporting 10 species; activity was recorded in five boxes during the sampling period using inspector cameras. Mitochondrial markers are indicated as

^*^COI; ^**≡**^ 12S–16S rRNA.

*^¤^* cyt b (also see associated figures). Arrows (↓) indicate whether the population is decreasing in size. Abbreviations shown in parentheses correspond to sampling locations.

ω IUCN status is provided at the species level; subspecies remain unassessed.

& See methods for further details.

The taxonomic assignment of bat species at each roosting site was based on a combination of at least two of three criteria: (i) Analysis of the mitochondrial markers COI, 12S–16S and cyt b present in the meta-transcriptomic data. These sequences were compared with the species reported for the visited locations and against the BOLD Identification System and NCBI databases (Ratnasingham et al. 2024); (ii) Historical occupancy records and prior survey reports based on morphological characteristics; (iii) Distribution data for known bat species in each site. The species identified in this study included the little forest bat (*V. vulturnus*), the lesser long-eared bat (*N. geoffroyi*), and the Southern bent-wing bat (*Miniopterus schreibersii bassanii*) (Table 1). Of note, only COI, 12S–16S and cyt b host markers were detected that were congruent with species records for the sampled sites. However, species identification was inherently biased due to the limited representation of Australian species in reference databases (e.g. Bold and NCBI). No additional sequencing was performed to further confirm host identity.

### Sample processing and sequencing

Bat faecal samples were thawed and homogenized by vortexing in the lysis reagent DNA/RNA shield at high speed for 30 seconds to ensure thorough mixing and shearing of the material. To facilitate the release of the nucleic acids and remove cellular debris, we used QIAshredder columns by centrifuging the samples at 4 °C at maximum speed for 3 minutes. Total RNA was extracted using the QIAamp Viral RNA Kit (Qiagen, AU) according to the manufacturer’s instructions and quantified using the Qubit fluorometer.

The extracted RNA was subjected to ribosomal depletion, reverse-transcribed into cDNA, and then used as input for paired-end library preparation using the Illumina Stranded Total RNA Prep with Ribo-Zero Plus (Illumina). Finally, paired-end libraries were sequenced on the Illumina NovaSeq X platform using the 300 cycle.

### Raw data processing and assembly

Paired-end raw data were preliminary assessed with FASTQC v.0.11.8 (Andrews 2010) to identify low-quality ends and the presence of adapter sequences. Subsequently, reads were trimmed to maintain a Phred score of 25. A second round of quality assessment was then performed with FASTQC. Contigs were *de novo* assembled using Megahit v.1.29 with default settings (Li et al. 2015). Relative contig abundance was calculated as the number of transcripts per million (TPM) and reads per million using RSEM v1.3.0 (Li and Dewey 2011) and Salmon v.0.8.2 (Patro et al. 2017). Cross-contamination between libraries due to index-hopping was considered likely when abundance values fell below 0.1% of the highest count recorded for a given contig across libraries.

### Contig annotation and taxonomic profiling

To identify potential virus sequences, contigs were initially screened against a database of RNA-dependent RNA polymerase (RdRp) sequences using the RdRp-Scan tool (Charon et al. 2022). These sequences were then compared against the nucleotide (NCBI-nt) and non-redundant (NCBI-nr) databases for taxonomic annotation and curation with e-value thresholds set to ≤1E-10 and ≤ 1E-4, respectively. Other virus gene fragments were identified using the protein Reference Viral DataBase (RVDB-prot) v28 database (Bigot et al. 2020). Open reading frames (ORFs) were predicted using the EMBOSS GetOrf tool (Rice et al. 2000), while domains and motifs were identified by scanning against all the Interpro databases using InterProScan v.5.63–95.0 (Jones et al. 2014). The taxonomic composition of libraries was assessed using Kraken v.2.1.1 (core-nt database v.04–2024) (Wood and Salzberg 2014) and MetaPhlan v.4.1.0 (Blanco-Míguez et al. 2023). To identify clusters of sequences originating from different geographic regions, amino acid contig sequences were clustered using CD-Hit v.4.8.1, with a sequence identity threshold of ≥98% to group highly similar sequences.

### Abundance and phylogenetic analysis

The phylogenetic relationships of the virus sequences identified in the microbat data in the context of related sequences publicly available on the NCBI/GenBank database were estimated using the maximum likelihood (ML) method available in IQ-TREE v.2.3.6 (Nguyen et al. 2015). In each case, the best-fit models of amino acid substitution for the RdRp sequences were assessed with the option -m TEST (Supplementary Table S1). Node support was estimated using the Shimodaira-Hasegawa approximate likelihood ratio test (SH-aLRT) and ultrafast bootstrap (UFBoot) support, with topological confidence set as SH-aLRT > = 80% and UFboot > = 95%, respectively. The likely host assignment for each virus, particularly whether they likely infected vertebrate or invertebrates (the latter being diet associated), was inferred from their phylogenetic relationships (i.e. vertebrate-associated viruses tend to form distinct clades), supplemented by metadata on sample origin (e.g. host, tissue type, geographic location), when available.

## Workflow and code availability

Most of the steps in this analytical procedure were followed as implemented in the Batch Artemis SRA Miner pipeline v. v1.0.4 (https://github.com/JonathonMifsud/BatchArtemisSRAMiner/tree/v.1.0.4), with adaptations made as needed.

## Results

### Mammalian-associated virus diversity in Australian microbats

To better understand the diversity and evolution of RNA viruses circulating in microbats in Australia, we sequenced 57 microbat faecal samples collected from several locations in New South Wales and South Australia (Table 1). When such information was available or based on direct observation of individuals, samples were taken from populations with individuals showing no overt signs of disease and no mortality events. Sequencing yielded between 36 million and 312 million paired-end reads per library. A total of 3 192 497 contigs were assembled, including 11 580 of viral origin (Fig. 1). Virus abundance varied between 0.01–215 530 TPM.

Despite the small sample size we identified a broad range of likely mammalian- and diet-associated viruses spanning nine taxonomic groups: the orders *Picornavirales* and *Bunyavirales*, and the families *Nodaviridae, Hepeviridae*, *Coronaviridae*, *Astroviridae*, *Chuviridae*, *Rhabdoviridae*, *Reoviridae* and *Astroviridae* (Fig. 1). Of these, picornaviruses were the most prevalent group in bat faecal samples (57/57 libraries), whereas hepeviruses and chuviruses were the least commonly detected (12/57 libraries) (Fig. 1). Approximately, 48.8% of the viruses discovered (n = 61) were likely mammalian in origin in that they were related to other mammalian-associated viruses on phylogenetic trees. Of note, 30 newly discovered viruses were identified in cave-roosting bats, while 95 newly discovered viruses were present in faecal samples collected from bat boxes. Prior to formal classification, we provisionally named the newly discovered viruses as ‘AusMicrobat’ viruses.

**Figure 2:**
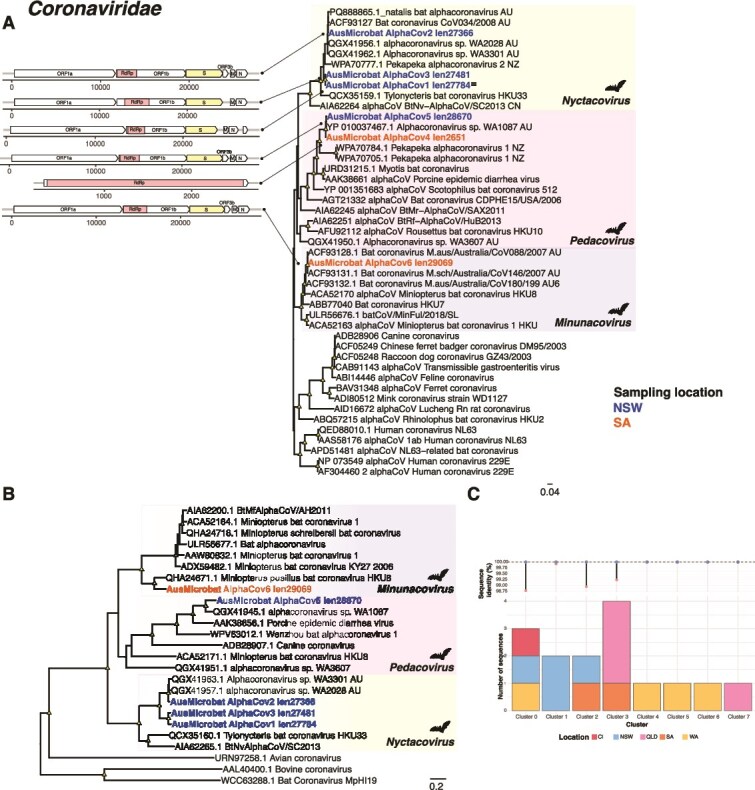
Phylogenetic relationships among the alphacoronaviruses identified in this study. Maximum likelihood trees are based on the amino acid sequences of the (A) RdRp—nsp12 and (B) spike protein. All phylogenetic trees are mid-point rooted for clarity only. Scale bars represent the number of amino acid substitutions per site. Known genera are represented by colour-coded clades for easier interpretation. Tip labels for novel viruses are coloured by sampling location—Blue (NSW), orange (SA). Nodal support values ≥80% SH-aLRT and ≥ 95% UFboot are denoted with yellow triangles at nodes. Bat-associated lineages are indicated with bat silhouettes. A schematic representation of the newly discovered alphacoronavirus sequences is displayed next to the tip labels, with annotated ORFs and the RdRp and spike domains shown in colour. (C) Clustering of alphacoronavirus sequences across multiple sampling locations in Australia. Each cluster comprises sequences sharing ≥98% amino acid sequence identity with the representative sequence. Australian locations are indicated as follows: CI (Christmas Island), NSW (new South Wales), WA (Western Australia), QLD (Queensland) and SA (South Australia). ^**≡**^ next to sequence names denotes those from libraries where 12S–16S transcripts were detected.

**Figure 3:**
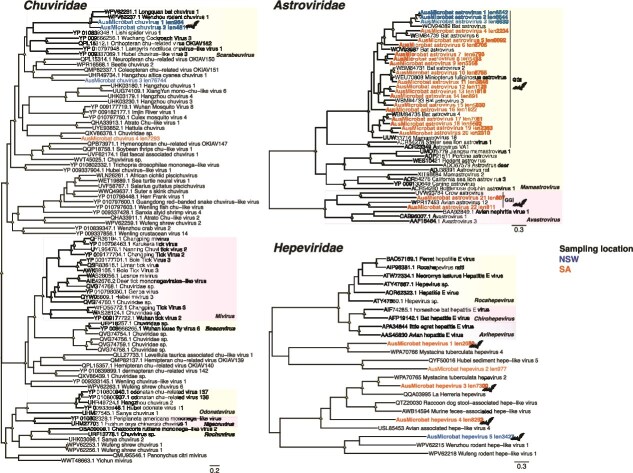
Phylogenetic relationships among the (A) *Chuviridae*, (B) *Astroviridae,* and (C) *Hepeviridae.* Trees are based on the amino acid sequences of the putative RdRp (*Chuviridae* and *Hepeviridae*) or capsid (*Astroviridae*). All phylogenetic trees are mid-point rooted for clarity only. Scale bars represent the number of amino acid substitutions per site. Known genera are represented by colour-coded clades for interpretation. Tip labels for novel viruses are coloured by sampling location—Blue (NSW), orange (SA). Nodal support values ≥80% SH-aLRT and ≥ 95% UFboot are denoted with yellow triangles at nodes. Tip labels highlighted in bold represent likely mammalian-associated lineages, also indicated with bat silhouettes.

The bat viruses identified here that were most closely related to known viruses belonged to the *Coronaviridae* (99.5% amino acid identity to known viruses in the RdRp) and the *Sedoreoviridae* (76%–99% amino acid identity to known viruses in the VP1) (Table 2). The novel astroviruses exhibited moderate to high similarity to other bat astroviruses (~60%–93% amino acid identity in the capsid), while some had similarity to avian astroviruses (~53% amino acid identity). The most genetically divergent virus sequences were also identified in the *Sedoreoviridae*, including AusMicrobat sedoreoviruses 1 and 8 (23%–36% identity to known viruses), and were likely diet-associated as they were unrelated to known vertebrate viruses. Viral abundance estimates indicated that reoviruses (TPM = 0.76–24 225) and picornaviruses (TPM = 0.16–2 200) were relatively abundant, while rhabdoviruses were the least abundant (TPM = 0.52–5.84). Also of note was that the viruses identified in the bat faecal samples collected from SA and NSW were often closely related. For example, highly similar coronaviruses (up to 96% genome-scale amino acid identity) and astroviruses (up to 93% amino acid identity in the capsid protein) were detected between both states, and which also clustered phylogenetically with viruses found in various locations in Australia as well as Christmas Island (a remote Australian territory located about 2 600 km from the mainland) (Figs 2 and 3).

**Table 2:** RNA viruses discovered in bat guano collected from sampling locations in NSW and SA, Australia. Taxonomic virus groups are indicated as well as the closest hit in the NCBI non-redundant (nr) database.

Virus	Family	Length	Accession code	Best hit (NCBI-nr)	% ID	E-value	Likely host
AusMicrobat AlphaCov1	*Coronaviridae*	27 784	QGX41962.1	polyprotein 1ab [Alphacoronavirus *sp.*]	81.0	0.0	Mammal
AusMicrobat AlphaCov2	*Coronaviridae*	27 366	YP_010799723.1	polyprotein 1ab [Alphacoronavirus *sp.*]	97.0	0.0	Mammal
AusMicrobat AlphaCov3	*Coronaviridae*	27 481	QGX41962.1	polyprotein 1ab [Alphacoronavirus *sp.*]	81.0	0.0	Mammal
AusMicrobat AlphaCov4	*Coronaviridae*	2651	YP_010037467.1	polyprotein 1ab [Alphacoronavirus *sp.*]	99.5	0.0	Mammal
AusMicrobat AlphaCov5	*Coronaviridae*	28 670	YP_010037467.1	polyprotein 1ab [Alphacoronavirus *sp.*]	96.9	0.0	Mammal
AusMicrobat AlphaCov6	*Coronaviridae*	29 069	WCZ55891.1	MAG: ORF1a protein [Miniopterus bat coronavirus HKU8]	73.4	0.0	Mammal
AusMicrobat chuvirus 1	*Chuviridae*	964	WPV62231.1	MAG: RNA-dependent RNA polymerase, partial [Longquan bat chuvirus 1]	80.9	72e-180	Mammal
AusMicrobat chuvirus 2	*Chuviridae*	4817	WNV56441.1	MAG: RNA-dependent RNA polymerase [Wenzhou rodent chuvirus 1]	65.3	0.0	Mammal
AusMicrobat chuvirus 3	*Chuviridae*	7674	UHR49734.1	MAG: RNA-dependent RNA polymerase [Hangzhou altica cyanea chuvirus 1]	42.6	0.0	Invertebrate
AusMicrobat chuvirus 4	*Chuviridae*	7293	WPR16568.1	MAG: RNA-dependent RNA polymerase, partial [Beetle chuvirus 2]	37.6	0.0	Invertebrate
AusMicrobat astrovirus 1	*Astroviridae*	6542	WNK76727.1	MAG: capsid precursor protein [Bat astrovirus]	61.0	61e-307	Mammal
AusMicrobat astrovirus 2	*Astroviridae*	6644	WNK76727.1	MAG: capsid precursor protein [Bat astrovirus]	61.0	4.98e-307	Mammal
AusMicrobat astrovirus 3	*Astroviridae*	6639	WNK76727.1	MAG: capsid precursor protein [Bat astrovirus]	61.0	5.66e-309	Mammal
AusMicrobat astrovirus 4	*Astroviridae*	2234	WNK76727.1	MAG: capsid precursor protein [Bat astrovirus]	58.8	6.91e-252	Mammal
AusMicrobat astrovirus 5	*Astroviridae*	6692	WBM84744.1	MAG: capsid protein, partial [Bat astrovirus 10]	84.7	0.0	Mammal
AusMicrobat astrovirus 6	*Astroviridae*	6706	XBH23922.1	MAG: capsid protein [Miniopterus bat astrovirus]	61.8	0.0	Mammal
AusMicrobat astrovirus 7	*Astroviridae*	6793	XBH23922.1	MAG: capsid protein [Miniopterus bat astrovirus]	75.3	0.0	Mammal
AusMicrobat astrovirus 8	*Astroviridae*	5483	XBH23922.1	MAG: capsid protein [Miniopterus bat astrovirus]	72.3	0.0	Mammal
AusMicrobat astrovirus 9	*Astroviridae*	5565	XBH23922.1	MAG: capsid protein [Miniopterus bat astrovirus]	74.0	0.0	Mammal
AusMicrobat astrovirus 10	*Astroviridae*	6768	WBM84731.1	MAG: capsid protein [Bat astrovirus 2]	92.9	0.0	Mammal
AusMicrobat astrovirus 11	*Astroviridae*	3646	WBM84731.1	MAG: capsid protein [Bat astrovirus 2]	85.3	0.0	Mammal
AusMicrobat astrovirus 12	*Astroviridae*	1128	WEU70808.1	MAG: capsid protein precursor, partial [Miniopterus fuliginosus astrovirus]	84.0	8.16e-210	Mammal
AusMicrobat astrovirus 13	*Astroviridae*	1818	WBM84733.1	MAG: capsid protein [Bat astrovirus 3]	75.0	5.59e-279	Mammal
AusMicrobat astrovirus 14	*Astroviridae*	891	WEU70808.1	MAG: capsid protein precursor, partial [Miniopterus fuliginosus astrovirus]	86.6	3.25e-173	Mammal
AusMicrobat astrovirus 15	*Astroviridae*	5830	WBM84733.1	MAG: capsid protein [Bat astrovirus 3]	75.1	0.0	Mammal
AusMicrobat astrovirus 16	*Astroviridae*	1822	UUW33716.1	ORF2, partial [Mamastrovirus 18]	69.7	3.83e-289	Mammal
AusMicrobat astrovirus 17	*Astroviridae*	7061	WBM84735.1	MAG: capsid protein [Bat astrovirus 4]	87.9	0.0	Mammal
AusMicrobat astrovirus 18	*Astroviridae*	6692	WBM84735.1	MAG: capsid protein [Bat astrovirus 4]	82.0	0.0	Mammal
AusMicrobat astrovirus 19	*Astroviridae*	2363	WBM84735.1	MAG: capsid protein [Bat astrovirus 4]	67.6	0.0	Mammal
AusMicrobat astrovirus 20	*Astroviridae*	2310	WBM84735.1	MAG: capsid protein [Bat astrovirus 4]	69.6	0.0	Mammal
AusMicrobat astrovirus 21	*Astroviridae*	807	UVW93784.1	capsid protein, partial [Crow astrovirus]	52.6	3.75e-68	Mammal
AusMicrobat astrovirus 22	*Astroviridae*	811	WPR17453.1	MAG: capsid protein [Avian astrovirus 12]	52.8	2.64e-85	Mammal
AusMicrobat hepevirus 1	*Hepeviridae*	2050	WPA70766.1	MAG: nonstructural polyprotein, partial [*Mystacina tuberculata* hepevirus 4]	45.2	6.86e-179	Mammal
AusMicrobat hepevirus 3	*Hepeviridae*	7300	QQA03995.1	polyprotein [La Herreria hepevirus]	41.3	0.0	Mammal
AusMicrobat hepevirus 4	*Hepeviridae*	8292	QQA03995.1	polyprotein [La Herreria hepevirus]	30.8	2.52e-195	Mammal
AusMicrobat hepevirus 5	*Hepeviridae*	3427	WPV62215.1	WPV62215.1 MAG: non-structural polyprotein, partial [Wenzhou rodent hepe-like virus 1]	34.0	4.87e-103	Mammal
AusMicrobat hepevirus 2	*Hepeviridae*	977	QYF50016.1	MAG: replicase, partial [Hubei sediment hepe-like virus 5]	37.2	1.44e-50	Invertebrate
AusMicrobat nodavirus 7	*Nodaviridae*	2707	WPA70719.1	MAG: RNA-dependent RNA polymerase, partial [*M. tuberculata* nodavirus 4]	42.5	1.37e-91	Mammal
AusMicrobat nodavirus 1	*Nodaviridae*	3265	QBS55246.1	RNA-dependent RNA polymerase [Tetranychus urticae-associated nodavirus A]	62.0	0.0	Invertebrate
AusMicrobat nodavirus 2	*Nodaviridae*	3410	QUS52660.1	RNA-dependent RNA polymerase [Mute swan faeces associated noda-like virus 2]	67.0	0.0	Invertebrate
AusMicrobat nodavirus 3	*Nodaviridae*	8454	WPV63042.1	MAG: RNA-dependent RNA polymerase [Wufeng bat nodavirus 1]	34.2	1.86e-138	Invertebrate
AusMicrobat nodavirus 4	*Nodaviridae*	4667	ADI48250.1	putative RdRp [Bat guano associated nodavirus GF-4n]	43.1	1.93e-215	Invertebrate
AusMicrobat nodavirus 5	*Nodaviridae*	2812	ASU47554.1	polymerase [Lone star tick nodavirus]	41.2	3.71e-173	Invertebrate
AusMicrobat nodavirus 6	*Nodaviridae*	3763	UQB76069.1	RNA-dependent RNA polymerase [Flumine noda-like virus 3]	39.1	5.30e-186	Invertebrate
AusMicrobat nodavirus 8	*Nodaviridae*	4993	XHA85947.1	MAG: putative RNA-dependent RNA polymerase [Qianjiang noda-like virus 29]	36.5	3.98e-95	Invertebrate
AusMicrobat nodavirus 9	*Nodaviridae*	5407	XHA85922.1	MAG: hypothetical protein [Qianjiang noda-like virus 20]	98.3	0.0	Invertebrate
AusMicrobat nodavirus 10	*Nodaviridae*	6478	WPV63071.1	MAG: RNA-dependent RNA polymerase [Wufeng shrew nodamuvirus 1]	38.4	9.20e-103	Invertebrate
AusMicrobat nodavirus 11	*Nodaviridae*	3092	WAX26160.1	MAG: RNA-dependent RNA polymerase [Army ant associated Nodavirus 1]	70.7	0.0	Invertebrate
AusMicrobat nodavirus 12	*Nodaviridae*	5532	WPA70725.1	MAG: RNA-dependent RNA polymerase [Bat nodavirus]	93.5	0.0	Invertebrate
							
AusMicrobat picornavirus 9	*Picornavirales*	5366	WPV63438.1	MAG: RNA-dependent RNA polymerase [Longquan rodent picorna-like virus 1]	54.3	0.0	Mammal
AusMicrobat picornavirus 10	*Picornavirales*	11 453	WFG77351.1	MAG: polyprotein [Bat picornavirus 5]	69.5	0.0	Mammal
AusMicrobat picornavirus 15	*Picornavirales*	7646	AEM23660.1	polyprotein [Bat picornavirus 2]	78.4	0.0	Mammal
AusMicrobat picornavirus 16	*Picornavirales*	7727	YP_004782528.1	polyprotein [Bat picornavirus 1]	79.1	0.0	Mammal
AusMicrobat picornavirus 1	*Picornavirales*	11 568	UDL13970.1	MAG: polyprotein [Xiangshan picorna-like virus 4]	30.2	0.0	Invertebrate
AusMicrobat picornavirus 2	*Picornavirales*	15 044	YP_009337161.1	hypothetical protein [Hubei picorna-like virus 27]	39.5	0.0	Invertebrate
AusMicrobat picornavirus 3	*Picornavirales*	10 580	YP_009110667.1	polyprotein [*Laodelphax striatellus* picorna-like virus 2]	66.8	0.0	Invertebrate
AusMicrobat picornavirus 4	*Picornavirales*	11 286	UDL13967.1	MAG: polyprotein [Xiangshan picorna-like virus 1]	30.5	0.0	Invertebrate
AusMicrobat picornavirus 5	*Picornavirales*	1319	WIW43234.1	MAG: putative polyprotein, partial [*Pteropus rufus* picorna-like virus]	92.9	1.22e-225	Invertebrate
AusMicrobat picornavirus 6	*Picornavirales*	6473	QKK82954.1	hypothetical protein, partial [*Erigeron annuus* picorna-like virus]	90.9	0.0	Invertebrate
AusMicrobat picornavirus 7	*Picornavirales*	9422	QZZ63320.1	hypothetical protein [Nelson Picorna-like virus 3]	74.5	0.0	Invertebrate
AusMicrobat picornavirus 8	*Picornavirales*	9857	UUV42601.1	MAG: polyprotein [Tuatara cloaca-associated picorna-like virus-1]	32.5	1.65e-269	Invertebrate
AusMicrobat picornavirus 11	*Picornavirales*	11 017	WPR16530.1	MAG: RNA-dependent RNA polymerase, partial [Avian associated picorna-like virus 41]	49.3	0.0	Invertebrate
AusMicrobat picornavirus 12	*Picornavirales*	1905	UYL83183.1	MAG: RNA-dependent RNA polymerase, partial [XiangYun tombus-noda-like virus 5]	51.6	1.67e-21	Invertebrate
AusMicrobat picornavirus 13	*Picornavirales*	2727	QUS52703.1	polyprotein [Mute swan faeces associated picorna-like virus 7]	31.0	5.15e-94	Invertebrate
AusMicrobat picornavirus 14	*Picornavirales*	6533	QED42981.1	hypothetical protein, partial [Pleurotus picornavirus A]	44.4	0.0	Invertebrate
AusMicrobat picornavirus 17	*Picornavirales*	11 194	WBM81658.1	MAG: RNA-dependent RNA polymerase [*Myotis brandtii* picorna-like virus 1]	31.7	2.78e-212	Invertebrate
AusMicrobat picornavirus 18	*Picornavirales*	6502	WPV63421.1	MAG: RNA-dependent RNA polymerase [Jingmen shrew picorna-like virus 1]	30.4	1.29e-284	Invertebrate
AusMicrobat picornavirus 19	*Picornavirales*	9276	UFZ21066.1	MAG: structural polyprotein [Planococcus ficus-associated picorna-like virus 1]	46.2	5.82e-209	Invertebrate
AusMicrobat picornavirus 20	*Picornavirales*	7967	WPV63450.1	MAG: RNA-dependent RNA polymerase, partial [Wenzhou bat picorna-like virus 8]	43.1	2.20e-56	Invertebrate
AusMicrobat picornavirus 21	*Picornavirales*	11 716	UXD80005.1	putative non-structural polyprotein [Lasius neglectus picorna-like virus 3]	46.6	5.11e-286	Invertebrate
AusMicrobat picornavirus 22	*Picornavirales*	10 855	YP_009336820.1	hypothetical protein [Hubei picorna-like virus 53]	30.0	0.0	Invertebrate
AusMicrobat picornavirus 23	*Picornavirales*	12 484	YP_009337118.1	hypothetical protein [Hubei picorna-like virus 52]	33.8	0.0	Invertebrate
AusMicrobat picornavirus 24	*Picornavirales*	12 831	UDL13977.1	MAG: RNA dependent RNA polymerase [Xiangshan picorna-like virus 7]	25.2	4.24e-130	Invertebrate
AusMicrobat picornavirus 25	*Picornavirales*	1997	AQY59901.1	RdRp, partial [Statovirus B1]	28.2	8.48e-46	Invertebrate
AusMicrobat picornavirus 26	*Picornavirales*	11 186	UQZ09572.1	putative polyprotein [Freshwater macrophyte associated picorna-like virus 12]	25.3	1.71e-98	Invertebrate
AusMicrobat picornavirus 27	*Picornavirales*	6340	XEQ84329.1	hypothetical protein [Mink picorna-like virus 1]	34.3	2.86e-123	Invertebrate
AusMicrobat picornavirus 28	*Picornavirales*	5594	UXD80013.1	putative non-structural polyprotein [*Myrmica rubra* picorna-like virus 4]	52.6	0.0	Invertebrate
AusMicrobat calicivirus 1	*Picornavirales: Caliciviridae*	6514	WAP91247.1	MAG: nonstructural polyprotein, partial [Avian associated calicivirus 5]	46.8	0.0	Mammal
AusMicrobat calicivirus 2	*Picornavirales: Caliciviridae*	10 043	WAP91247.1	MAG: nonstructural polyprotein, partial [Avian associated calicivirus 5]	46.7	0.0	Mammal
AusMicrobat calicivirus 3	*Picornavirales: Caliciviridae*	10 146	USL85468.1	MAG: non-structural polyprotein [Avian associated calicivirus 1]	39.2	0.0	Mammal
AusMicrobat rhabdovirus 3	*Rhabdoviridae*	1205	WWV88395.1	MAG: L protein, partial [Rhabdovirus *sp.*]	71.6	5.49e-201	Mammal
AusMicrobat rhabdovirus 4	*Rhabdoviridae*	2488	WWV88387.1	MAG: L protein [Rhabdovirus *sp.*]	65.9	0.0	Mammal
AusMicrobat rhabdovirus 12	*Rhabdoviridae*	3510	WPV62811.1	MAG: RNA-dependent RNA polymerase [Wenzhou bat rhabdovirus 2]	72.2	0.0	Mammal
AusMicrobat rhabdovirus 13	*Rhabdoviridae*	2288	WPV62809.1	MAG: putative glycoprotein [Wenzhou bat rhabdovirus 2]	68.4	3.88e-113	Mammal
AusMicrobat rhabdovirus 1	*Rhabdoviridae*	770	WPV62848.1	MAG: RNA-dependent RNA polymerase, partial [Wufeng shrew rhabdovirus 15]	59.4	5.49e-90	Invertebrate
AusMicrobat rhabdovirus 2	*Rhabdoviridae*	764	QQP18754.1	RNA-dependent RNA polymerase [Soybean thrips rhabdo-like virus 2]	52.7	2.70e-82	Invertebrate
AusMicrobat rhabdovirus 5	*Rhabdoviridae*	13 603	YP_009204560.1	L protein [Fox faecal rhabdovirus]	33.5	8.48e-256	Invertebrate
AusMicrobat rhabdovirus 6	*Rhabdoviridae*	1415	QMP82309.1	RNA-dependent RNA polymerase, partial [Hemipteran rhabdo-related virus OKIAV26]	57.6	5.05e-169	Invertebrate
AusMicrobat rhabdovirus 7	*Rhabdoviridae*	14 451	QMP82194.1	RNA-dependent RNA polymerase [Coleopteran rhabdo-related virus OKIAV28	34.0	0.0	Invertebrate
AusMicrobat rhabdovirus 8	*Rhabdoviridae*	13 467	WGF21055.1	L [*Spodoptera frugiperda* rhabdovirus] [*S. frugiperda* rhabdovirus]	82.9	0.0	Invertebrate
AusMicrobat rhabdovirus 9	*Rhabdoviridae*	9821	YP_010798566.1	RNA-dependent RNA polymerase [Lepidopteran rhabdo-related virus 34]	47.4	0.0	Invertebrate
AusMicrobat rhabdovirus 10	*Rhabdoviridae*	1040	DAZ87777.1	TPA_asm: hypothetical protein [Metorhabdovirus 1]	78.0	3.63e-180	Invertebrate
AusMicrobat rhabdovirus 11	*Rhabdoviridae*	1480	DAZ87842.1	TPA_asm: hypothetical protein [Clonorhabdovirus 1]	77.9	7.18e-263	Invertebrate
AusMicrobat phenuivirus 1	*Bunyavirales*	1976	WPV62188.1	MAG: RNA-dependent RNA polymerase [Wufeng shrew phenuivirus 9]	78.1	0.0	Mammal
AusMicrobat phenuivirus 2	*Bunyavirales*	1140	WPV62186.1	MAG: RNA-dependent RNA polymerase [Wufeng shrew phenuivirus 7]	69.4	1.19e-173	Mammal
AusMicrobat phenuivirus 10	*Bunyavirales*	741	WPV62168.1	MAG: RNA-dependent RNA polymerase [Wenzhou bat phenuivirus 2]	60.3	4.24e-94	Mammal
AusMicrobat phenuivirus 3	*Bunyavirales*	702	DBA56514.1	TPA_asm: RNA-dependent RNA polymerase [*Neotermes castaneus* phenuivirus 1	51.3	1.06e-63	Invertebrate
AusMicrobat phenuivirus 4	*Bunyavirales*	850	QOR29577.1	RNA-dependent RNA polymerase, partial [Bat bunyavirus]	57.9	1.03e-99	Invertebrate
AusMicrobat phenuivirus 5	*Bunyavirales*	1541	APL98134.1	RNA-dependent RNA polymerase, partial [Bat bunyavirus JTM]	56.2	1.47e-174	Invertebrate
AusMicrobat phenuivirus 6	*Bunyavirales*	7039	UVZ34188.1	RNA-dependent RNA polymerase [Erthesina fullo bunyavirus 1]	39.5	0.0	Invertebrate
AusMicrobat phenuivirus 7	*Bunyavirales*	7570	QMP82296.1	RNA-dependent RNA polymerase [Lepidopteran phenui-related virus OKIAV270]	58.4	0.0	Invertebrate
AusMicrobat phenuivirus 8	*Bunyavirales*	9004	XGY11997.1	MAG: RNA-dependent RNA polymerase [Empoasca fabae phenuivirus 1]	42.5	1.90e-212	Invertebrate
AusMicrobat phenuivirus 9	*Bunyavirales*	6996	QMP82311.1	RNA-dependent RNA polymerase [Blattodean phenui-related virus OKIAV261]	40.9	0.0	Invertebrate
AusMicrobat phenuivirus 11	*Bunyavirales*	989	XCN99621.1	RNA-dependent RNA polymerase [Switchgrass phenui-like virus 1]	59.6	6.01e-130	Invertebrate
AusMicrobat bunyavirus 1	*Bunyavirales*	794	WPV62125.1	MAG: RNA-dependent RNA polymerase [Jingmen bat bunyavirus 1]	75.8	1.10e-126	Mammalian
AusMicrobat bunyavirus 2	*Bunyavirales*	2868	AOA33722.1	putative RNA dependent RNA polymerase [Crithidia otongatchiensis leishbunyavirus CotoLBV1]	49.8	4.92e-289	Invertebrate
AusMicrobat bunyavirus 3	*Bunyavirales*	1881	UPT53676.1	MAG: RNA-dependent RNA polymerase [*Bactrocera correcta* trypanosomatid leishbunyavirus]	56.5	4.98e-234	Invertebrate
AusMicrobat bunyavirus 4	*Bunyavirales*	1439	APG79301.1	RNA-dependent RNA polymerase, partial [Hubei bunya-like virus 5]	45.3	8.94e-124	Invertebrate
AusMicrobat bunyavirus 5	*Bunyavirales*	7763	DBA56512.1	TPA_asm: RNA-dependent RNA polymerase [*N. castaneus* bunya-like virus 1]	39.2	0.0	Invertebrate
AusMicrobat sedoreovirus 1	*Sedoreoviridae*	4013	WPV63639.1	MAG: RNA-dependent RNA polymerase, partial [Jingmen bat reo-like virus 1]	36.40	1.48e-241	Mammal
AusMicrobat sedoreovirus 2	*Sedoreoviridae*	3991	APG79114.1	RdRp [Hubei reo-like virus 12]	36.70	2.08e-223	Mammal
AusMicrobat sedoreovirus 3	*Sedoreoviridae*	2031	AVM87459.1	RNA-dependent RNA polymerase [Wenling scaldfish reovirus]	26.20	2.04e-57	Mammal
AusMicrobat sedoreovirus 4	*Sedoreoviridae*	2655	XBH23973.1	MAG: VP1 protein, partial [Mops bat reovirus]	46.70	3.11e-272	Mammal
AusMicrobat sedoreovirus 5	*Sedoreoviridae*	636	QCX36715.1	structural protein VP1, partial [Human rotavirus]	99.10	3.27e-139	Mammal
AusMicrobat sedoreovirus 6	*Sedoreoviridae*	3640	UUW33709.1	VP1 protein [Rotavirus J]	92.70	0.0	Mammal
AusMicrobat sedoreovirus 7	*Sedoreoviridae*	731	AWV67027.1	VP1, partial [Bat rotavirus H]	75.60	7.90e-116	Mammal
AusMicrobat sedoreovirus 7	*Sedoreoviridae*	1979	AWV67027.1	VP1, partial [Bat rotavirus H]	82.10	0.0	Mammal
AusMicrobat sedoreovirus 7	*Sedoreoviridae*	6118	AWV67027.1	VP1, partial [Bat rotavirus H]	81.00	0.0	Mammal
AusMicrobat sedoreovirus 7	*Sedoreoviridae*	5485	AWV67027.1	VP1, partial [Bat rotavirus H]	81.10	0.0	Mammal
AusMicrobat sedoreovirus 7	*Sedoreoviridae*	11 121	AWV67027.1	VP1, partial [Bat rotavirus H]	81.00	0.0	Mammal
AusMicrobat sedoreovirus 8	*Sedoreoviridae*	4253	WPV63640.1	MAG: RNA-dependent RNA polymerase [Jingmen bat reo-like virus 2]	23.10	9.08e-55	Invertebrate
AusMicrobat sedoreovirus 8	*Sedoreoviridae*	4254	WPV63640.1	MAG: RNA-dependent RNA polymerase [Jingmen bat reo-like virus 2]	23.10	9.10e-55	Invertebrate
AusMicrobat sedoreovirus 9	*Sedoreoviridae*	4258	UOI84724.1	RNA-dependent RNA polymerase [*Ceratitis capitata* reo-like virus 1]	33.10	3.78e-188	Invertebrate
AusMicrobat sedoreovirus 10	*Sedoreoviridae*	1608	APG79196.1	RNA-dependent RNA polymerase [Hubei reo-like virus 6]	34.70	2.13e-83	Invertebrate
AusMicrobat sedoreovirus 11	*Sedoreoviridae*	2000	APG79200.1	RdRp [Hubei reo-like virus 5]	23.70	3.49e-23	Invertebrate
AusMicrobat sedoreovirus 12	*Sedoreoviridae*	1930	APG79166.1	RdRp [Hubei reo-like virus 9]	25.40	8.70e-29	Invertebrate

**Figure 4:**
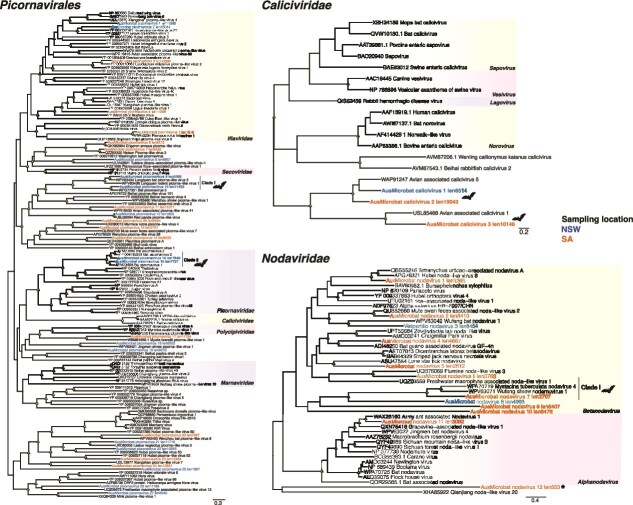
Phylogenetic relationships among the (A) *Picornavirales* (B) *Caliciviridae*, and (C) *Nodaviridae*. Trees are based on the amino acid sequences of the putative RdRp. All phylogenetic trees are mid-point rooted for clarity only. Scale bars represent the number of amino acid substitutions per site. Known genera are represented by colour-coded clades for easier interpretation. Tip labels for novel viruses are coloured by sampling location—Blue (NSW), orange (SA). Nodal support values ≥80% SH-aLRT and ≥ 95% UFboot are denoted with yellow triangles at nodes. Tip labels highlighted in bold represent likely mammalian-associated lineages, also indicated with bat silhouettes. ^*^ Next to sequence names denotes those from libraries where COI transcripts were detected.

**Figure 5:**
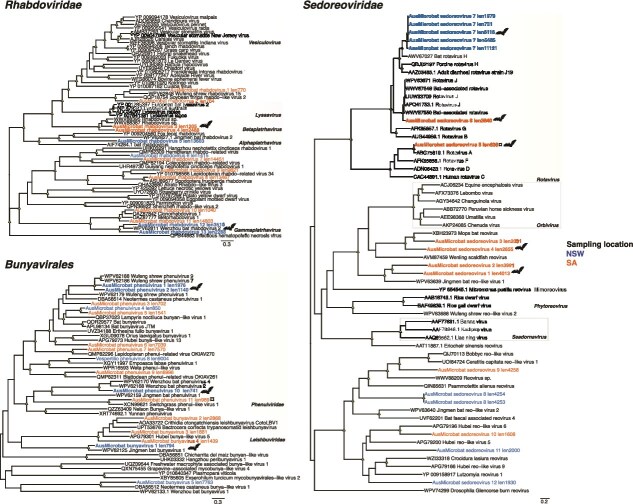
Phylogenetic relationships among the (A) *Bunyavirales* and (B) *Sedoreoviridae*. Trees are based on the amino acid sequences of the putative RdRp. All phylogenetic trees are mid-point rooted for clarity only. Scale bars represent the number of amino acid substitutions per site. Known genera are represented by colour-coded clades for easier interpretation. Tip labels for novel viruses are coloured by sampling location – Blue (NSW), orange (SA). Nodal support values ≥80% SH-aLRT and ≥ 95% UFboot are denoted with yellow triangles at nodes. Tip labels highlighted in bold represent likely mammalian-associated lineages, also indicated with bat silhouettes. *¤* next to sequence names denotes those from libraries where cyt b transcripts were detected.

To investigate the potential co-circulation of viruses at a single location, we focused on the virus profile found in bat faecal samples collected from the Naracoorte bat maternity caves as it houses a single bat species. Accordingly, we detected diverse mammalian-associated viruses co-circulating in the Southern bent-wing bat population, including a high diversity of mamastroviruses, but also rhabdoviruses, bunyaviruses, coronaviruses, hepeviruses, nodaviruses, picornaviruses and reoviruses shed in bat guano (Figs. 2–5).

### RNA virus families

#### Coronaviridae

A striking result of this study was the relatively large number (six) of alphacoronaviruses identified in microbat faecal samples from NSW and SA. In marked contrast, no betacoronaviruses were identified in our sampling. The bat alphacoronaviruses identified here fell into three subgenera: *Nyctacovirus*, *Minunacovirus*, and *Pedacovirus* (Fig. 2A, B)*.* As expected, the genome sequences of the newly identified alphacoronaviruses ranged from 27.36–32.02 Kb and exhibited the genomic configurations expected for these viruses. The coronaviruses detected in faecal samples from NSW were highly abundant (TPM = 13.2–418.43) in comparison to those found in SA samples (TPM = 0.23–3.4), perhaps reflecting local aspects of virus ecology or sampling artefacts. Of note, based on their phylogenetic placement and sequence identity, the newly identified members of the subgenus *Nyctacovirus* were closely related (97.2%–99.77% amino acid identity in the RdRp) to those found in different microbat species sampled in Western Australian, Queensland, as well as Christmas Island, and hence indicative of a broad geographic distribution, although due to limited size no direct ecological inferences can be drawn. Similarly, the alphacoronavirus sequences from the subgenus *Pedacovirus* were closely related to an alphacoronavirus identified in Western Australia (Fig. 2A, B). AusMicrobat alphacoronavirus 6 (TPM = 3.4) from the subgenus *Minunacovirus* was closely related to sequences circulating in Queensland and Southern Asia, including *Miniopterus* bat coronavirus HKU8 previously detected in bat faecal samples from Hong Kong (97.6% amino acid identity in the RdRp) and associated with rhinolopid bats (Fig. 2A-B) (Smith et al. 2016). These geographic patterns were consistent with the clustering analysis, in which clusters contained sequences from various locations across Australia, including NSW and SA (Fig. 2C).

#### Chuviridae

Also of significance was the identification of four chuviruses (*Chuviridae*) in microbat faeces collected from bat boxes in NSW (n = 3) and SA (n = 1) and provisionally termed AusMicrobat chuviruses 1–4. These sequences shared up to 78% amino acid identity among themselves and grouped with viruses from the genus *Scarabeuvirus* as well as with unclassified chuviruses (TPM = 0.34–5.75) (Fig. 3A). Of most note, in our phylogenetic analysis AusMicrobat chuvirus 1–2 (TPM = 0.34–0.52) clustered with Longquan bat chuvirus 1 and Wenzhou rodent chuvirus 1 (~92%–95% amino acid identity in the RdRp), previously sampled from the gut and lung of the Chinese rufous horseshoe bat (*Rhinolophus sinicus*) and the striped field mouse (*Apodemus agrarius*) [42], respectively. Together, these four sequences formed a distinct mammalian lineage within the *Chuviridae*. As such, this provides strong evidence for the sustained transmission of exogenous chuviruses in mammals on multiple continents. In contrast, AusMicrobat chuvirus 3 grouped with invertebrate-associated viruses primarily detected in coleopterans, while AusMicrobat chuvirus 4 formed a lineage with a viral sequence identified in an avian faecal metagenome, indicative of a dietary association.

#### Astroviridae

We identified a high diversity of astroviruses (n = 22) that clustered with bat viruses within the genus *Mamastrovirus* (TPM = 0.46–138), with genomes ranging from ~ 5–7 kb in length. The majority of these viruses were detected in samples from the Naracoorte maternity caves in SA (91–98% amino acid identity) and were related to sequences found in intestine, skin, and swabs samples from *Miniopterus spp.* (*Miniopteridae*) from Australia (NSW) and China (59–93% amino acid identity). The remaining mamastroviruses formed a clade comprising sequences from NSW (TPM = 0.46–1.29), which grouped with a Chinese sequence detected in the intestine of a *Pipistrellus pipistrellus* bat. Finally, two other novel astroviruses were detected at low abundance levels and grouped within the genus *Avastrovirus* (TPM = 0.08–0.18) (Fig. 3B), primarily associated with infection in birds (~53% amino acid identity). Whether this reflects cross-species transmission between bats and birds is uncertain.

#### Hepeviridae

The majority of the newly discovered hepeviruses were related to those found in vertebrate metagenomes (Fig. 3C), suggesting a mammalian origin. This was exemplified by AusMicrobat hepevirus 5 (TPM = 0.38) which grouped with rodent hepeviruses in our phylogenetic analysis. Notably, two of the novel hepeviruses from the SA samples were related to viruses found in bat guano from lesser short-tailed bats (*Mystacina tuberculata*) (TPM = 0.18–9.24), one of the only two native terrestrial mammals in New Zealand (31–63% amino acid identity). This could reflect either shared arthropod diets between Australian and New Zealand bats or virus-host co-divergence reflecting the separation of Australian and New Zealand bats some 16–18 million years ago [28, 43]. In contrast, AusMicrobat hepevirus 2 grouped with a virus previously identified in sediment in China (19% aa identity).

#### Picornavirales

We identified 30 viral contigs distributed among different families within the order *Picornavirales* (Fig. 4A), including the *Picornaviridae*, *Caliciviridae*, *Marnaviridae*, *Polycipiviridae*, *Iflaviridae* as well as previously unclassified viruses. The novel viruses exhibited amino acid identities to their closest relatives ranging from 25–93% in the RdRp (Table 2). Phylogenetic analysis indicated that AusMicrobat picornaviruses 9, 10, 15, and 16 were likely associated with mammals, grouping with sequences identified in faeces from bats and rodents (TPM = 7.37–2 199) (Fig. 4A; Clade II–II), whereas the remaining virus sequences were grouped with invertebrate-associated viruses and hence are likely dietary associated. Similarly, we identified three caliciviruses from samples collected in NSW and SA that shared 17–52% amino acid sequence identity between them in the RdRp (Fig. 4B). Only one calicivirus sequence was identified in a bat box from NSW (TPM = 0.8). However, the novel caliciviruses did not cluster with bat caliciviruses but instead formed a sister clade with avian caliciviruses (up to 39% RdRp amino acid identity) (Fig. 4B).

#### Nodaviridae

We identified a large number of nodavirus sequences (2707–8 454 bp) from bat faecal samples that were related to invertebrate viruses within the genus *Alphanodavirus*, sharing between 36–70% amino acid sequence identity in the RdRp with their closest relatives (Fig. 4C). However, AusMicrobat nodavirus 7 (TPM = 0.77) fell within a mammalian-related clade grouping with bat and shrew metagenome-derived sequences (Clade I), including viral sequences found in the New Zealand lesser short-tailed bat (*M. tuberculata*) and the Asian grey shrew (*Crocidura attenuate*), indicating both shared common ancestry and a likely mammalian origin.

#### Rhabdoviridae

We detected 13 novel rhabdoviruses, including five likely bat-associated viruses related to mammalian-derived sequences from bats and shrews (Fig. 5**A**). The newly identified AusMicrobat rhabdoviruses shared between 33–83% amino acid identity in the RdRp with their closest relatives, whereas their relative abundance levels ranged from 0.52–5.84 TPM (Table 2). In particular, the likely bat-associated viruses were related to metagenome-derived sequences previously identified from bats in China within the *Alpha*, *Beta* and *Gammaplatrhavirus* genera. For instance, AusMicrobat rhabdoviruses 3–4 (TPM = 1.08–1.26) formed a lineage with bat metagenome-derived sequences from the intestine and faecal samples of *Miniopterus pusillus* and *Hipposideros armiger* in China (66–72% amino acid identity in the RdRp), suggesting a broad circulation of rhabdoviruses in bats. Finally, invertebrate-associated rhabdoviruses were related to viral sequences detected in invertebrates as well as plants.

#### Bunyavirales

A total of 16 bunyaviruses were found in bat faecal samples collected from NSW and SA, with RdRp amino acid identities of 39–83% to already documented bunyavirus sequences (TPM = 0.11–760.29). Newly discovered bunyaviruses fell within diverse lineages from the families *Phenuiviridae* and *Leishbuviridae,* as well as a number of unclassified lineages (Fig. 5B)*.* AusMicrobat phenuiviruses 1, 2 and 10, as well as AusMicrobat bunyavirus 1 formed distinct clades with vertebrate metagenome-derived sequences, including bats and shrews, indicating either a mammalian origin or the potential to infect mammalian hosts. For example, AusMicrobat phenuivirus 10 (TPM = 0.44) grouped with viral sequences previously identified *Myotis laniger* and *Myotis ricketti* (60% amino acid identity in the RdRp) within the *Phenuiviridae*, strongly suggestive of a bat origin. The remaining viruses (n = 12) were related to invertebrate-associated sequences, including arthropods and protozoan trypanosomatids (Fig. 5A, Table 2).

#### Reoviridae

Twelve reovirus sequences belonging to the family *Sedoreoviridae* were identified, of which nine grouped with viruses previously detected in bat samples, with amino acid identities ranging from 23%–92% in the VP1 (RdRp) to their closest relatives (Fig. 5B). AusMicrobat reoviruses 5–7 fell within the genus *Rotavirus* (TPM = 0.12–24 225), grouping with members of the species *Rotavirus A*, *Rotavirus H* and *Rotavirus J*, which are typically mammalian viruses. Of these, AusMicrobat reovirus 6 was found at high abundance in Southern bent-wing bats from the Naracoorte caves, SA (TPM = 24 225), from which we were able to identify 10 out of 11 segments (encoding the VP1, VP3, VP4, VP6, VP7, NSP1, NSP2, NSP3, NSP4 and NSP5 proteins). Similarly, AusMicrobat reoviruses 1–4 clustered with viruses found in metagenome-derived sequences from bats and fish. Conversely, AusMicrobat reoviruses 10–12 were more closely related to invertebrate-associated viral sequences corresponding to unclassified reoviruses.

## Discussion

Bats have long been considered important reservoirs for mammalian related viruses of zoonotic potential (Fagre and Kading 2019, Wang et al. 2023, Liu et al. 2024). Herein, we employed total RNA sequencing on bat faecal samples collected from both natural and artificial roosting sites to identify RNA viral families that are likely to infect mammals (Wu et al. 2012, Wang et al. 2023). As expected, most of the viruses (~51.2%) detected were of dietary (i.e. reflecting prey composition) or parasite (e.g. leishbuviruses) origin, reflecting from the presence of arthropods and plants present in bat droppings (Li et al. 2010, Wu et al. 2016). However, despite the small sample size, we also detected a high number of likely mammalian-associated viruses, which seemingly exhibited greater viral richness and abundance than that previously observed in metatranscriptomic studies of viruses in Australian megabats (Ng and Baker 2013, Van Brussel et al. 2023, Ortiz-Baez et al. 2025). These findings align with those of previous studies that have identified that a broad range of RNA viruses circulate in microbats (Wu et al. 2016, Salmier et al. 2017, Wang et al. 2023) (Table 2, Fig. 1).

Of particular note was the identification of a novel chuvirus in Australian bats that was closely related to those found in bats and rodents from China, and hence which represents the occurrence of a mammalian-specific chuvirus lineage (as it is untenable that the same dietary-associated viruses would appear in bats on different continents). Although chuviruses were originally described as invertebrate-associated, and the detection of AusMicrobat chuvirus 3–4 likely reflects prey composition, there is growing evidence for a broader host range (Shi et al. 2018, Chen et al. 2023, Laovechprasit et al. 2024, Mélade et al. 2025). Indeed, chuvirus-like sequences have been detected in faecal samples and tissues of bats, rodents, marsupials, and shrews (Chen et al. 2023, Harvey et al. 2023) although they did not form a distinct lineage as documented here. The close phylogenetic relationship of AusMicrobat chuvirus 1–2 with Longquan bat chuvirus 1 and Wenzhou rodent chuvirus 1 (~92%–95% amino acid identity in the RdRp) strongly suggests that this particular lineage of chuviruses is circulating in mammals on multiple continents (Fig. 3) (Chen et al. 2023). This observation is consistent with the recent isolation of an infectious chu-like virus from tumour cells from the Tasmanian devil (*Sarcophilus harrisii*) indicative of active virus replication in mammalian cells (Mélade et al. 2025) (although this virus lineage is very different to that detected here). More detailed studies of distribution and biology of chuviruses in mammals are clearly warranted.

In a similar fashion to the chuviruses, the clustering of AusMicrobat nodavirus 7 with vertebrate-host derived sequences from Australia, New Zealand and China (Fig. 4) implies a potential association with bats (Bennett et al. 2019). Members of the *Nodaviridae* are primarily associated with invertebrates and fish within the *Alphanodavirus* and *Betanodavirus* genera, respectively. However, the discovery of alphanodaviruses with multi-organ distribution such as the porcine nodavirus and bat nodavirus present in brain tissues from pigs and bats, respectively, bolsters the idea that they might also be associated with mammalian infection (Dacheux et al. 2014, Chen et al. 2023, Orf et al. 2023). Future detection of the novel microbat nodavirus (or its relatives) in bat tissues would provide additional support their association with mammalian infection.

Viruses of likely bat-origin were also identified in a number of other virus families, including the *Coronaviridae*, *Astroviridae*, and *Reoviridae*. With respect to the coronaviruses, we identified bat-associated viruses belonging to three subgenera of *Alphacoronavirus* and related to sequences detected in bats from various geographic locations across Australia, including the Naracoorte caves in South Australia (Figs. 1, 2). The high diversity of astroviruses found in the Naracoorte bat caves suggests that the high population density and close contact of Southern bent-wing bats (i.e. roosting ecology) supports their ongoing transmission and evolution. Indeed, as the Naracoorte bat caves harbour a large maternity colony (~20 000 to 35 000 individuals), maternal care behaviour might facilitate the circulation of astroviruses from mothers to offspring, particularly given the immature immune systems of newborns and juvenile bats and the extended periods spent together during nursing (Mendenhall et al. 2017). These findings align with research on shedding pulses and high levels of astrovirus genetic diversity circulating in cave-roosting bats (Zhu et al. 2009, Horigan et al. 2024). Similarly, the detection of viral sequences related to rotaviruses A and J (Fig. 5) suggests that these cave-dwelling bats harbour a high diversity of rotaviruses.

The data generated here also revealed the geographic connectivity of coronaviruses and caliciviruses between NSW and SA based on the phylogenetic clustering of virus sequences across sampling locations. Of note most, we observed a high diversity and widespread circulation of alphacoronaviruses in microbats within Australia. This was reflected in the phylogenetic clustering of viruses from NSW and South Australia (Fig. 2A, B), which were similarly closely related to sequences from Queensland, Western Australia and Christmas Island (Fig. 2A–C). This is indicative of the historical and ecological connectivity (i.e. shared evolutionary history and habitat similarities) of bat populations within Australia and, sporadically, with locations as distant as Christmas Island (Prada et al. 2019). In addition, our data provided evidence for virus transmission between bat species. A striking example was the phylogenetic clustering and high sequence similarity of virus sequences found in the little forest bat with those from the large-footed myotis (*Myotis macropus*) and the Christmas Island flying fox (*Pteropus natalis*), suggesting relatively recent cross-species transmission (Prada et al. 2019, Wang et al. 2023). Similarly, the identification of shared caliciviruses between distant roosting locations suggests that microbat populations in the region maintain connectivity through migration, likely sharing seasonal sites, foraging and staging areas (Fig. 1). Indeed, radio tracking research on southern bent-wing bats from the Naracoorte bat caves showed females flying around 25–35 Km on commuting night flights (Bourne 2010), while monitoring studies of banded bats recaptured Naracoorte bats in Wombeyan, NSW, over 1 000 Km apart (Hamilton-Smith 1970). Similar findings have been observed for coronaviruses circulating among *Chalinolobus spp.* from Western Australia (Prada et al. 2019). Notably, the co-circulation of AusMicrobat calicivirus 1–3 suggests a hidden calicivirus diversity in Lesser long-eared bat populations in SA (Fig. 4).

In other instances, the data generated here supported far longer virus-host associations, including the possibility of virus-host co-divergence on evolutionary time-scales. This was most apparent in the case of the bat hepeviruses identified here that were most closely related to those previously found in lesser short-tailed bats from New Zealand that diverged from their Australian relatives some 16–18 million years ago (Hand et al. 1998, Waller et al. 2024) (Fig. 3). Similar findings have been documented for coronaviruses shared between Australian and New Zealand microbats (Waller et al. 2024). However, formal assessment of this interpretation will require co-phylogenetic and molecular clock analyses based on larger data sets that are beyond the scope of the present study. In addition, many of the novel viruses grouped with relatives previously identified in multiple microbat species. This was particularly evident in the *Astroviridae*, *Rhabdoviridae*, *Coronaviridae*, and *Chuviridae*. For instance, virus sequences from the southern bent-wing bat and classified within the *Rhabdoviridae* (AusMicrobat rhabdovirus 3–4) and *Astroviridae* (AusMicrobat astrovirus 10–11), were related to those circulating in *Miniopterus spp.*, suggesting that host-jumping and a broader virus host-range might be commonplace (Hayman 2016, Dacheux et al. 2014).

While this study has expanded our knowledge of virus diversity, it says little about their disease potential. As a case in point, although astroviruses are typically associated with gastroenteritis disease in humans, and have been in found microbat skin lesions (Van Brussel et al. 2023), previous research suggest that these viruses can circulate in apparently healthy bats (Chu et al. 2008). As the health status of the sampled Southern bent-wing bat colony in the Naracoorte bat caves is unknown, further studies are needed to determine whether astroviruses represent a pathogenic threat for this critically endangered species (Mendenhall et al. 2015, Van Brussel et al. 2023). Similarly, relatives of the novel AusMicrobat reoviruses, including Rotavirus A (RVA) and H (RVH), are known to circulate in mammals and birds, including humans, livestock, and bats (Kim et al. 2016, Simsek et al. 2021). Although these cause gastroenteritis disease in some species, it is unclear whether they are pathogenic in bats and the risk they pose for zoonotic transmission (Yinda et al. 2018, Simsek et al. 2021). Previous studies have also revealed RVA cross-species transmission events between humans and bats (Asano et al. 2016, He et al. 2017, Simsek et al. 2021). Of note, as *Miniopterus schreibersii* has a widespread distribution and a propensity to roost with various other bat species (Bányai et al. 2017) it is important to determine whether the circulation of the novel AusMicrobat rotaviruses poses a potential health threat.

An important limitation of our study was the inability to establish host–virus associations due to the nature of the roost-level guano sampling and the limited taxonomic representation of bats in public databases. With the exception of the known presence of the Southern bent wing bat in the Naracoorte caves, the roosting sites were shared by multiple bat species, preventing definitive links between detected viruses and their bat hosts. Although this study offers insights into the likely mammalian-associated RNA virome of microbats, further tissue sampling and follow-up molecular investigations are required to establish host-virus associations, the occurrence of co-infections, and the pathogenic potential of the viruses identified. Indeed, because only a subset of viruses are excreted in guano, the virome composition in these samples necessarily differs from that found in bat tissues (Van Brussel et al. 2023) In addition, the incorporation of bat ectoparasite sampling from roosting sites would help to determine whether they act as viral vectors, both within and between bat species (Szentiványi et al. 2024). More broadly, because our approach is minimally invasive, longitudinal sampling could be implemented to better understand the effects of environmental conditions, seasonality and roost-species composition on the distribution and prevalence of the viruses observed.

This study highlights the high RNA viral diversity in microbats, while the identification of a lineage of mammalian origin in the *Chuviridae* provides an important insight into host-range evolution of these viruses. More broadly, these data further emphasize the ecological and geographical interconnectivity between Australian microbat populations as well as the occurrence of cross-species viral transmission. Future research should address the role of seasonality and roosting habitats in shaping virus circulation and patterns of cross-species transmission among microbats.

## Supplementary Material

Ortiz-Baez_Supplementary_Table_S1_veag017

## Data Availability

The raw sequence reads generated in this study are available at the NCBI SRA database under BioProject PRJNA626677 accessions SAMN49708426—SAMN49708445 and SAMN49893252—SAMN49893288. Virus consensus sequences have been deposited in the NCBI/GenBank database under accession numbers PX703778-PX703903.
