# Supplementary material for: Viral diversity, ecological interconnectedness, and the identification of mammalian chuviruses in Australian microbats
Source: Virus Evol. 2026 Mar 21;12(1):veag017. doi: 10.1093/ve/veag017 (PMC13070566; doi:10.1093/ve/veag017)
Supplement: Ortiz-Baez_Supplementary_Table_S1_veag017 [file ortiz-baez_supplementary_table_s1_veag017.docx]

**Supplementary Table S1.** Amino acid substitution models used in the phylogenetic analysis.

| Group | Model | Protein(s) |
| --- | --- | --- |
| *Astroviridae* | Q.pfam+F+I+Γ_4_ | Capsid |
| *Coronaviridae* | LG+F+I+Γ_4_ \| WAG+F+Γ_4_ | RdRp \| Spike |
| *Rhabdoviridae* | Q.pfam+F+I+ Γ_4_ | RdRp |
| *Bunyavirales* | Q.pfam+F+I+Γ_4_ | RdRp |
| *Caliciviridae* | Q.pfam+F+I+Γ_4_ | RdRp |
| *Chuviridae* | Q.pfam+F+I+Γ_4_ | RdRp |
| *Hepeviridae* | Q.pfam+F+I+Γ_4_ | RdRp |
| *Nodaviridae* | Q.pfam+F+I+Γ_4_ | RdRp |
| *Picornavirales* | Q.pfam+F+I+Γ_4_ | RdRp |
| *Reoviridae* | VT+F+I+Γ_4_ | RdRp |
